# Descriptive Mappings of Global-Related Research Studies on Invertebrates in the Context of Agriculture

**DOI:** 10.1155/tswj/7571101

**Published:** 2024-12-17

**Authors:** Emrobowansan Monday Idamokoro, Augustine Suh Niba

**Affiliations:** Department of Biological and Environmental Sciences, Faculty of Natural Sciences, Walter Sisulu University, Nelson Mandela Drive Campus, P/Bag X1, Mthatha 5117, Eastern Cape, South Africa

**Keywords:** bibliometric, farming, global trend, invertebrates, long-term research

## Abstract

Invertebrates form a vital component of agricultural ecosystems, and they are chief actors in sustaining the functions of the ecosystem and soil health. Scholarly publications that concentrated on visualizing the research outputs and trends on invertebrates and agriculture are scarce. In this paper, we adopted a bibliometric model to extract trends/research studies on invertebrates and agriculture between 1991 and 2022, using scholarly studies retrieved from the Web of Science (WoS) databank. Therefore, the aim of the study is to assess and analyse publications and findings on research studies/trends on invertebrates and agriculture. A total of 1201 articles were recovered from the WoS databank with average citations per doc and coauthors per document ratio of 31.22 and 4.79, respectively. Studies on invertebrates and agriculture research studies were positively correlated with the number of years (*R*^2^ = 0.7803; *y* = 3.4661*x* − 19.659) signifying an upsurge in the amount of publications on this topic in the near future. The United States maintained a top position in terms of published outputs (*n* = 312) and citations (*n* = 14,113), followed by Germany (*n* = 75; *n* = 3686) and the United Kingdom (*n* = 70; *n* = 3117), respectively. Articles from the United States (*n* = 67) and China (*n* = 32) had strong networks with other nations of the world. Top subject priorities in this research field in terms of author keywords are agriculture (*n* = 141), biodiversity (*n* = 66), arthropods (*n* = 66) and biological control/ecosystem services (*n* = 46). From our findings, economically stable nations such as the United States, Germany, China, the United Kingdom and Australia are carrying out more research on this subject matter compared to the developing countries. We also found out that from the thematic evolution and literature results, invertebrate research in the context of agriculture is tending towards biogeography, farmland biodiversity, insecticides and organic agriculture, which are of immense importance to scientists and researchers in this research domain, thus signifying the direction/path of future research.

## 1. Introduction

Agriculture and its related fields (including aquaculture, fisheries, crop production, pasture and forestry) face the problem of safeguarding the food security of a global growing human population with an estimation of about 3 billion more people (of its over 7 billion inhabitants) by the year 2050 [1]. It has been projected that this will necessitate more than a 60% rise in global food production [2]. With the knowledge that agriculture provides about 98% or more of the global food resource, and the increasing rate of the human population (about 1% annually), sustainable agricultural practices are essential to promote food security and poverty reduction. Assessing the viability of agriculture as a sector, and thereby forecasting the future situation of food security, there is the need for prior knowledge of how the agricultural systems work and the complex associations/relationships that occur among ecological biodiversity.

Invertebrates form a vital component of agricultural ecosystems, and they are chief actors in sustaining ecosystem functions and soil health. They are known to be significant agricultural biodiversity involved in regulating the strata and primary functions of the natural ecosystem [3]. Invertebrate species are small animals without backbones, and they are known to be principal in the natural food webs because they are part of the ecosystem engineers linked with agriculture [4]. As ecosystem engineers, they execute important physical roles in sustaining soil structure and processes [5]. They also maintain a key impact on agricultural productivity and hence are involved in a major role in food security [6]. Invertebrates play multifunctional roles in the natural ecosystem, which help improve agriculture by acting as agents of pollination and organic matter decomposition [7, 8]. Furthermore, soil invertebrates take part in critical soil processes that retain healthy productive soils and fertility [4].

Conversely, various groups of invertebrates have been reported to provide biological control of crop pests [9]. In several instances, invertebrates including *Cycloneda limbifer*, *Trichogramma* spp. and *Amblyseius nicholsi* among others form the basis for integrated pest management (IPM) strategy [9, 10]. Additionally, pollination activities by invertebrates are among the most reported and significant processes that structure ecological environments in both natural and agricultural settings [5, 6]. A projected figure of 60%–90% of the globe's flowering plants/crops relies on insects (including invertebrates) for pollination [4]. However, because this service (pollination) by invertebrates is seen to attract no financial cost, their pollinating activities receive little or no attention in agricultural management.

Some species of invertebrates are also used as food, while in other sectors, their products (e.g., honey and silk production) are of other economic use [4, 11, 12]. In some parts of the world, invertebrates are an important part of daily nutrition [13, 14], especially in the developing nations. For instance, invertebrates used for food include shell-fish, such as crustaceans (shrimp and lobsters) and mollusc (oysters), honey from bees, Cephalopod ink in the Italian and Spanish dishes (and other European nations), such as black pasta and squid in ink soup, and the Echinoderms, sea urchins and sea cucumbers [15]. The cultivation and utilization of invertebrates for consumption may appear as an ecologically standard source of animal protein for several people around the globe [11, 16]. In addition, invertebrates can be used as a nutrient cycler and as detritivores (i.e., detritus shredders, bioturbators and detritus grazers) in soils [17, 18]. Invariably, invertebrates play an important role in agricultural systems, and they possess the potential to be utilized to benefit agricultural production and at large improve food security [19].

Several scientific publications on the use of invertebrates as a tool to enhance agriculture have been reported [6, 8, 20]. However, we are unaware of any study that has employed the method of bibliometrics to report the research trends on invertebrates in the context of agriculture. Therefore, the scope of the current paper is to reveal publications on global research findings on the research studies/trends of invertebrates as linked to agriculture. The research question in relation to the present study is to first identify the knowledge base of research studies done on invertebrates in the context of agriculture. In addition, the research question for the study is to produce a global social network structure of related research studies on invertebrates and agriculture. Our study also pin-pointed the global research scope on the studies of invertebrates and agriculture, for example, authors, distribution of countries, research outputs, keywords, the worldwide trends of citation and trending topics on the subject of discussion.

This study hopes to systematically evaluate the global progressions and trends in the use of the aforementioned research field from a scholarly perspective using different bibliometric indexes. Bibliometric is an innovative tool that permutates the use of mathematical metrics and statistical matrix to map and systematically explain research happenings in a particular field [21–23].

Using bibliometric assessment to explain the significance of invertebrates in agriculture allows the current study to identify the key themes and subthemes within this research niche that will promote and improve agricultural production. Furthermore, identifying the influential research and significant investigations of our bibliometric assessment on this research area will help in translating scholarly knowledge into practical applications as well as innovations in the field of agriculture. Novel areas of research (for example, the use of genetic engineering in economically valuable insects) could be further studied and investigated, which could be critical for future improvements in agriculture. Also with the employment of the bibliometric tool, we may be able to leverage deep insights for various stakeholders such as scientists, organizations and policymakers into how to effectively address the challenges as well as the opportunities in sustainable agriculture, which in the long run will contribute to global food security as well as environmental health.

The current bibliometric study just like every other bibliometric study will help generate a knowledge structure capable of projecting future directions and hints on informative studies, thereby giving a broad view to researchers, all stakeholders and policymakers to strategize future inclinations and the dynamics involved in the multifacet relationships in research associated with invertebrates and agriculture for boosting the agricultural sector.

Summarily, the objectives of the present study include the following:

1. To describe the thematic structure and evaluation mapping of the field of invertebrates and agriculture,
2. To recognize the major research producers/influencers (i.e., nations, organizations and institutions) and
3. To compare the impact of their research publications and citations.

## 2. Methodology

### 2.1. Data Retrieval

The current paper employed the use of scientific articles on invertebrates as linked to agriculture research outputs, which were obtained from the Web of Science (WoS) database because this databank generates quality standard academic work. Again, WoS database is known to host reliable and efficient high-impact scholarly publications [24–27]. Furthermore, the WoS database was selected because it is reported to have a high volume of research publications on both physical and biological sciences [28, 29]. The advanced search command in WoS was adopted in our study due to the fact that it permits the building of long and comprehensive search questions. The common approach on the use of one database to perform bibliometric study is due to the fact that it is difficult to do them with multiple databases which may result in the loss of some relevant articles on the subject matter [30].

### 2.2. Search Strategy Adopted for Data Retrieval

In the present study, we created a search question that covers most of the associated number of publications with the slightest false-positive outcome by using Wikipedia after a thorough literature search on the research topic, particularly on related studies and systematic reviews to acquaint ourselves with the most appropriate keywords linked with the search topic [31, 32]. For a wider and broader search, we adopted the use of the topic search methodology for associated keywords on ‘invertebrates' and ‘agriculture' to retrieve all the needed documents for the current study. Gathering the associated/related data from databases requires a query search. The query entails searching for both keywords and Boolean functions/operations. Subsequently, the current work focuses on invertebrates in the context of agriculture; ‘agriculture', ‘farming' and ‘invertebrates' were the keywords of the query. The operator AND separates these keywords. Each of these has synonymous keywords that the authors frequently use in the field. The operator OR is used to separate these keywords within each group. The keywords include the agriculture keyword group (‘agriculture' and ‘farming') and the invertebrate keyword group (‘Coral', ‘Cnidar', ‘Octopus', ‘Squid', ‘Mollusc', ‘Velvet worm', ‘Onychophor', ‘Nautil', ‘crab', ‘Arthropod', ‘jellyfish', ‘echinoderm', ‘sponge' and ‘invertebrate'). Meanwhile, the Boolean operators AND and OR are utilized to constrain the scope of the resulting topics. Our study gathered all scientific research types, such as research papers, review papers, technical notes, book chapters, books, patents and conference proceedings published from 1991 to 2022 (the past three decades) from the WoS database. However, our study excluded all other research types but only included research paper types. Our final collected dataset of papers contains a sum of 1201 unique articles/documents.

### 2.3. Search Question Adopted for Data Utilization

An inclusive search question containing precise phrases that are associated with invertebrates as linked to agriculture was entered into the topic search engine of the WoS database, followed by detailed terms as a constraint to minimize and remove unwanted publications that will not contribute to the goal of the current study. In addition, our search in the WoS database was further refined to remove review articles, technical notes, proceedings, and other languages that is not English, e.g., Spanish and Japanese.

**Figure 1:**
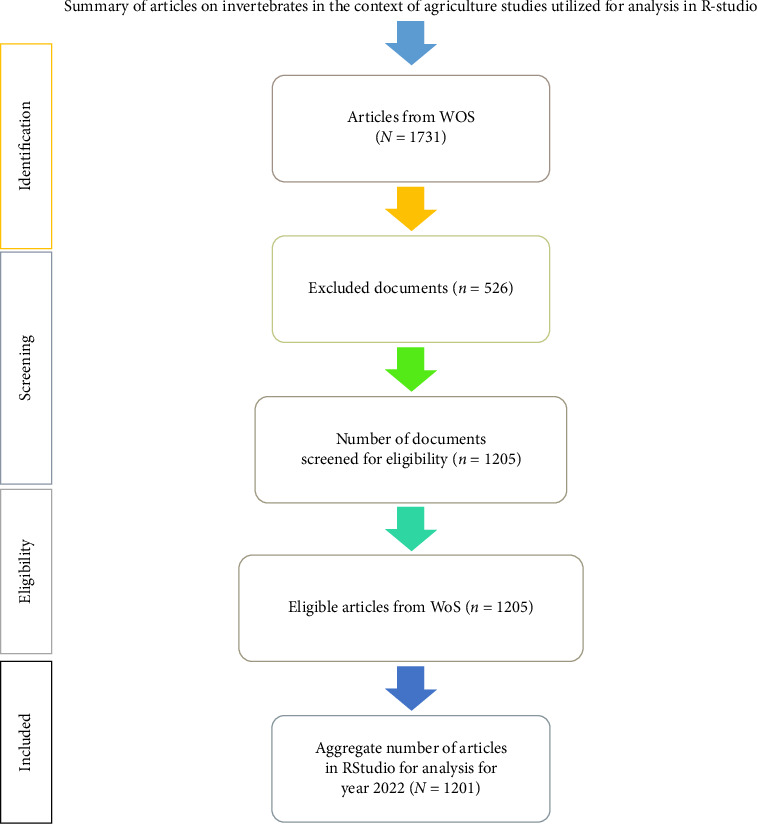
PRISMA flowchart for invertebrates in the context of agriculture documents.

The reason for excluding certain documents (e.g., proceeding papers and review articles) was not really to disregard the significance of these documents in academic dialog. While proceeding papers and review articles (as well as other publications) are known to contribute to the academic discourse and intellectual knowledge pool, their inclusion in the present bibliometric study can introduce inconsistencies as well as potentially skew outcomes and results. Conversely, excluding the aforementioned types of publications gives room for a more precise, consistent and focused assessment of what we intended our research findings to be, which will, in turn, lead to more reliable insights and conclusions of our findings. The search protocol and data treatment are well explained in Figure 1. The search queries for WoS that were employed are given below:

1250 results from WoS Core Collection for

Agriculture⁣^∗^ OR farming AND (Coral⁣^∗^ OR Cnidar⁣^∗^ OR Octopus⁣^∗^ OR Squid⁣^∗^ OR Mollusc⁣^∗^ OR ‘Velvet worm⁣^∗^' OR Onychophor⁣^∗^ OR Nautil⁣^∗^ OR crab⁣^∗^ OR Arthropod⁣^∗^ OR jellyfish⁣^∗^ OR echinoderm⁣^∗^ OR sponge⁣^∗^ OR invertebrat) (Topic).

Refined By: NOT Document Types: Proceeding Paper or Review Article or Book Chapters or Early Access. Click to remove this refine from your search. NOT Document Types: Editorial Material or Data Paper or Note or Item About an Individual. Click to remove this refine from your search. NOT Publication Years: 2023.

1214 results from Web of Science Core Collection for

Agriculture⁣^∗^ OR farming AND (Coral⁣^∗^ OR Cnidar⁣^∗^ OR Octopus⁣^∗^ OR Squid⁣^∗^ OR Mollusc⁣^∗^ OR ‘Velvet worm⁣^∗^' OR Onychophor⁣^∗^ OR Nautil⁣^∗^ OR crab⁣^∗^ OR Arthropod⁣^∗^ OR jellyfish⁣^∗^ OR echinoderm⁣^∗^ OR sponge⁣^∗^ OR invertebrat) (Topic).

Refined By: NOT Publication Years: 2023. Click to remove this refine from your search. NOT Document Types: Retracted Publication or Item About an Individual or Data Paper or Note or Early Access or Editorial Material or Book Chapters or Proceeding Paper or Review Article. Click to remove this refine from your search. NOT Languages: Russian or Dutch or Chinese or French or Portuguese or German or Spanish. Click to remove this refine from your search.

### 2.4. Data Processing and Analysis

The present work analysed the data obtained from WoS by using R version 4.3.0 (2023-04-21 ucrt) RStudio software together with Bibliometrix R package for bibliometric explanations [21]. All data retrieved were imported into RStudio for further analysis and visualization of results [33, 34]. Bibliometrix R package was used to retrieve data by utilizing models of the R package (R-project web interface in Biblioshiny) to describe our outcomes including citation analysis, author's keywords, authors' scientific performance, nations performance, and scholarly collaborations by nations and authors among others. This approach makes use of the RStudio package to deduce the outcomes (such as citation numbers, authors' impact, authors' collaborations and institution networks) and bibliometric evaluation of diagrammatic pairing (e.g., cocitation and keyword co-occurrences) of bipartite interactions of the rectangular indexes of research outputs × attributes. For example, the statistical description of a typical bibliometric network is elucidated as follows:

(1) $$\begin{array}{c} \text{Network}\left(N\right)=G\times H^{T}, \end{array}$$

where *G* represents a bipartite complex matrix of research publications × attributes (e.g., keywords plus, organization, countries, keywords, nations influence and publication citations). *N* signifies the symmetrical matrix *N* = *H*^*T*^.

Conversely, we employed an illustrative model for all networks with the use of a mathematical (software) language called the force-directed Fruchterman procedures, which was entered into the networkPlot command of the Bibliometrix R package. In addition, all the network links that were computed were normalized by Salton's cosine coefficient, proximity indexes (collaboration strength), Simpson's coefficient and Jaccard's similarity indexes among clusters of a network as described by Aria and Cuccurullo [21]. Again, the k-means clusters/nodes were carried out strictly on authors' keywords so as to evaluate ideas and concepts in invertebrate studies as linked to agriculture by using the function of theoretical model of the Bibliometrix R package as reported by Ref. [35].

## 3. Results

A total of 1201 publications were retrieved for the analysis within the studied time (1991–2022). The features of all the retrieved articles are summarized in Table 1. The number of authors for the entire study span was 4985, while other informetrics for the discussed subject matter comprises 69 single authors, 4.79% coauthors per document, 32.22% international coauthorships, 31.22% average citations per document, 56,346 total references and 11.03% annual growth rate of this research field .

**Figure 2:**
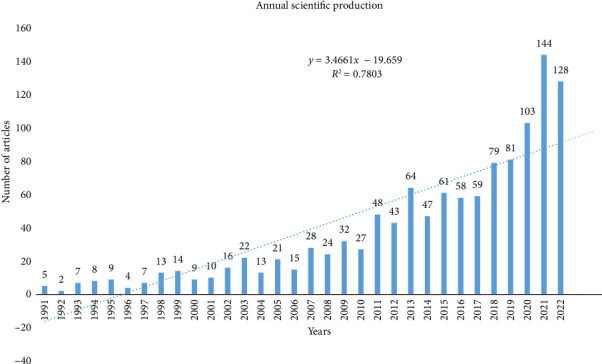
The annual number of articles on invertebrate and agriculture research from 1991 to 2022.

In addition, Figure 2 shows the result of data that were analysed using a polynomial function fitting curve. As shown in Figure 2, the polynomial model fitting evaluated by the yearly growth rate of research work showed a significant positive correlation (*R*^2^ = 0.7803; *y* = 3.4661*x* − 19.659) between the cumulative amount of articles and years of research publication. Figure 2 result also depicts a trend in publications that showed that research productions had some fluctuations between 1991 and 2015 during the survey period but with an improved rise in article production on invertebrates and agriculture from 2016 to 2021. The yearly growth rate of article production on invertebrates and agriculture research is 11.03%. The highest article number on the subject matter was recorded in 2021 (*n* = 144).

Table 2 shows the research publications on invertebrate and agriculture research for the top 25 most productive/relevant nations based on the number of outputs. The United States ranked first in the number of articles published in the field (*n* = 312; % of articles by a nation per total global articles = 25.97), followed by China (*n* = 88; % of articles by a nation per total global articles = 7.32), Germany (*n* = 75; % of articles by a nation per total global articles = 6.24), the United Kingdom (*n* = 70; % of articles by a nation per total global articles = 5.82) and Australia (*n* = 60; % of articles by a nation per total global articles = 4.99). The frequency of publications varied among the 25 topmost nations from 0.008 to 0.26. Again, the peak nations among the 25 globally placed countries with multiple-country publications (MCPs) were the United States (*n* = 67), China (*n* = 32), Germany (*n* = 32) and the United Kingdom (*n* = 31). The nations ranked in top positions for single-country publications (SCPs) of article outputs are the United States (*n* = 245), China (*n* = 56), Germany (*n* = 43) and the United Kingdom (*n* = 39) (Table 2).

**Figure 3:**
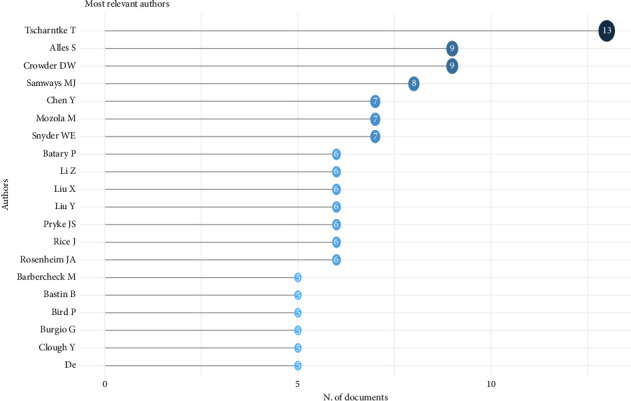
Top 20 relevant authors in the field of invertebrates and agriculture research.

Conversely, the most cited nations in invertebrates and agriculture research showed that the United States was placed in the first position (*n* = 14,113), while Germany (*n* = 3686), the United Kingdom (*n* = 3117), Australia (*n* = 1607) and Canada (*n* = 1419) lead the top chart (Table 3). From Figure 3, the result showed the 20 topmost relevant authors in the research niche of invertebrates and agriculture. The author by name T. Tscharntke was ranked first (*n* = 13), followed by S, Alles (*n* = 9), DW Crowder (*n* = 9), MJ Samways (*n* = 8) and Y Chen (*n* = 7). Based on the 20 topmost relevant keywords (as per author's keywords) by scientists in the field of invertebrates and agriculture research, it was observed that agriculture (*n* = 141) was ranked first, followed by biodiversity (*n* = 66), arthropod/s (*n* = 66), biological control (*n* = 46), ecosystem services (*n* = 46), sustainable agriculture (*n* = 33) and Carabidae (*n* = 31), among other keywords used by authors (Table 4).

The results for the top 25 journal sources with the greatest published outputs in the field of invertebrates linked with agriculture are enumerated in Table 5. These journal source include Agriculture Ecosystems and Environment (*n* = 69; 5.74%), Journal of AOAC International (*n* = 27; 2.24%), PLoS One (*n* = 25; 2.08%), Journal of Applied Ecology (*n* = 23; 1.91%) and Insects Journal (*n* = 21; 1.74%). Furthermore, the topmost prolific research organizations with more than 16 research articles are displayed in Table 6. The Michigan State University in the United States (number of articles = 56) maintained the first position; The University of California, Davis, in the United States was placed in the second position (number of articles = 39), while the University of Florida in the United States was placed in the third (number of articles = 38) and the University of Queensland in Australia was placed in the fourth position (number of articles = 35).

Table 7 indicates 20 topmost globally cited papers in research done on invertebrates as linked with agriculture based on the total number of citations from 1991 to 2022. The scholarly paper authored by S.R. Carpenter in the journal Ecological Application was placed in the first position with an aggregate of 3900 citations. The paper rated second was published by Sparks and Nauen in the journal Pest Biochemistry and Physiology with a total of 696 citations. The third (*n* = 527) and fourth (*n* = 466) highly cited journals had S. Seibold and T.G. Benton as authors, respectively (Table 7).

**Figure 4:**
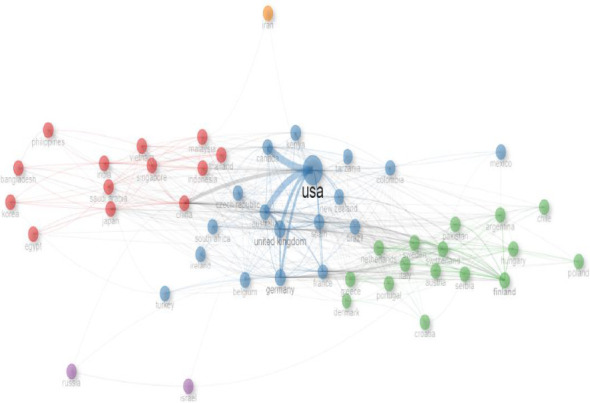
Collaborative mappings of networks of nations on research done on invertebrates and agriculture. Each node in the collaborative network is an individual nation and the diameter of the node corresponds to the number of publications. The lines/strokes denote the paths of networking between countries, and the thickness of lines/strokes signifies the degree of collaboration between the countries, while the five different colours seen in this figure represent the collaboration cluster of the nations.

Furthermore, Figure 4 shows the network/collaboration visualization map of nations' co-operation, depicting nations that had at least six articles. The single node in the network is a distinct nation, and the diameter/distance of the node agrees to the amount of publications by an individual nation. The strikes depict the direction of networking between nations, and the thickness of strikes/strokes shows the extent of collaboration between the nations. The five different colours (green, orange, blue, purple and red) symbolize the networking grouping of the nations. Collaboration links ranged from 2 to 25. The United States had the highest number of collaborations (*n* = 67), followed by China (*n* = 32), Germany (*n* = 31) and the United Kingdom (*n* = 31).

**Figure 5:**
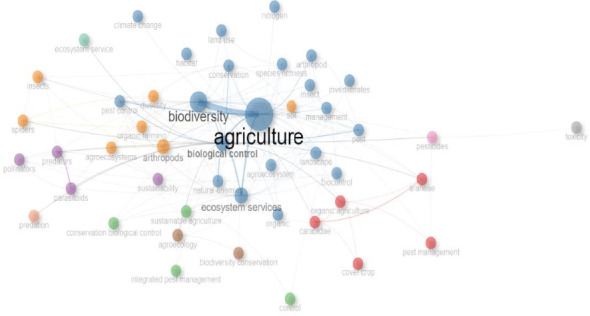
Keyword networking and association strength of worldwide studies on invertebrates and agriculture. Each node in the network shows the individual keywords, and the diameter of the node correlates with other keyword strengths. Lines/strokes represent the links of association between keywords, while the different colours observed in this figure depict the collaboration cluster of the keywords.

In the same vein, Figure 5 shows the co-occurrence collaboration/network and interrelationship of the 50 topmost terminologies on invertebrate and agriculture research, which are displayed in a pictographic web. Each of the circles with different colours denotes a cluster of terminology, and the networking strides/strokes depict the level of collaboration with regard to keywords. Furthermore, the level of proximity the keywords are to each other, the more the likelihood of their closeness in the scientific literature during the study period of 1991–2022. The network visualization of frequently occurring keywords primarily shows the regularly utilized words in invertebrate and agriculture research, which makes it easier to differentiate the niche areas of focus in this field.

**Figure 6:**
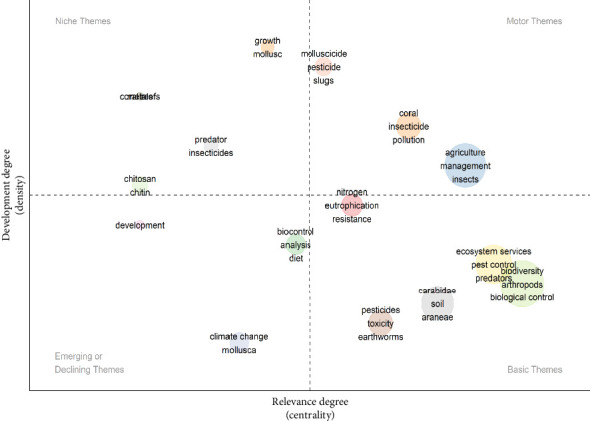
Thematic map (author keywords) in the research niche of invertebrates as linked to agriculture.

Figure 6 examines the authors' keywords via the thematic evaluation map. This result represents four key themes/topics found on the authors' keywords collaboration and clustering: the motor theme, the niche theme, basic theme and the emerging theme. From these four themes, the authors' keywords such as ‘Molluscicide', ‘pesticide', ‘coral', ‘insecticides', ‘agriculture', management', ‘insects', ‘growth', ‘mollusc', ‘coral reefs', ‘predator', ‘chitin', chitosan', ‘nitrogen', ‘eutropication', ‘resistance', ‘pesticides', ‘toxicity', ‘earthworm', ‘carabidae', ‘soil', ‘araneae', ‘ecosystem services', ‘pest control', ‘biodiversity', ‘arthropods' and ‘biological controls', ‘biocontrol', ‘diet', ‘climate change', ‘mollusca' and ‘development' were the focus in the field of invertebrate and agriculture, among others.

**Figure 7:**
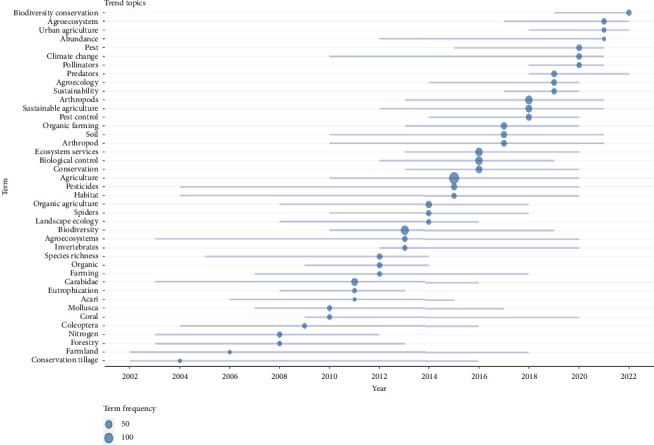
Top trending topics of author keywords on invertebrates and agriculture research studies with high-frequency terms over between 50 and 100.

Furthermore, the result from Figure 7 analysed the contents of the author keywords, which gives readers a rich and current understanding of the publication trends and outlines perhaps some limitations of the invertebrate and agriculture study field. This result excerpts the subject trends of the literature items by promoting better keywords from the analysis of the means of the subject matter in RStudio. The line denotes the timeline of a topic, and the radius of the circle is proportional to the number/amount of articles that trail a topic trend. The darker the intensity of the circle's colour, the higher the number of mentions/citations that topic trend attracts. To be represented in this graph (Figure 7), the topic frequency must exceed 25 times. The trend started in 2004 only with the ‘conservation tillage'. The trend of topic then evolves to represent the associated studies in invertebrate and agriculture studies over the years. Top trending topics of author keywords on invertebrates and agriculture research studies with high-frequency terms over the years include agriculture, arthropods, biological control, biodiversity and ecosystem services. These aforementioned author keywords top the chart with high terminology frequencies of between 50 and 100 from 2004 to 2022.

## 4. Discussions

The present study analysed the scientific publications on research studies on invertebrates from the perspective of agriculture and its significant trends between 1991 and 2022 based on the data retrieved from the WoS database. The amount of research publications on the studied field grew in a nonlinear manner from five articles in 1991 to 128 articles in 2022. There were visible fluctuations in the rate of article outputs from the year 1996 to 2016. However, there was a steady surge in publications on the studied subject matter from 2017 to 2022, indicating increasing interest in studies on invertebrates as linked to agriculture research in the last three decades. This improved observation of article numbers over the years may probably be due to the continual effort of scientists and researchers to explore innovative avenues of advancing the use of invertebrates to promote agriculture. Several researchers have reported studies on the use of invertebrates as pollinators, decomposers, food, predators and biological control agents [4, 6, 36–39]. Invertebrates are a major part of the ecological food webs, and they form part of the bionetwork/bioenvironmental engineers linked with agriculture [6]. According to Ref. [4], invertebrate species have a key influence on agricultural productivity and therefore participate in key roles in ensuring food security. Again, the yearly increase in the scholarly production graph in Figure 2 pinpoints the fact that scientific outputs on invertebrates and their use for improving agriculture are growing fast, which further suggests a future increase.

Figure 2 displays the hierarchy of invertebrate research as linked to agriculture with a total number of research work written on a yearly basis. Various studies have been done on different aspects of the use of invertebrates to improve agriculture with the highest number of publications recorded in the year 2021 with about 144 research articles followed by the year 2022 with the total number of publications of 128 research articles during the study period, while the years 1991 and 1992 have five and two research articles published, respectively. Studies on invertebrates related to agriculture research during the study period experienced a rise from early 2010 and peaking in 2021 followed by 2022 with a Kolmogorov–Smirnoff goodness-of-fit of 0.7803. Conversely, research output on invertebrates and agriculture research fluctuated mostly during the survey period between 1996 and 2017; for instance, the result shows that the research studies published on invertebrates and agriculture in years 2004, 2005, 2006, 2007, 2008 and 2009 are 13, 21, 15, 28, 24 and 32 articles, respectively.

As commonly reported in other bibliometric studies, large numbers of relevant authors involved in the use of invertebrate species to promote agriculture were mainly from advanced nations such as the United States, China, Germany, the United Kingdom, Australia and Canada, among others. The financial and economic stability of advanced nations contributes to their continuous exploration in advancing research in various scientific fields for the purpose of combating multifaceted societal challenges that confront humans [40, 41]. Only South Africa (an African nation) was observed (Table 2) to be part of other global countries linked with doing research on invertebrates as linked to agriculture. This observation may be quite instructive and a pointer to other developing nations (and especially African countries) who may want to key in to this field of research area. The current situation of food shortage/agricultural challenges experienced in some developing countries and mostly in sub-Saharan African nations should motivate more scientists in these regions to explore the prospects of doing research with regard to the studied subject matter. Since proven evidence in the use of invertebrates to advance agriculture and food production has been successful over the years [6, 37–39], adopting these research areas may also be encouraged in other nations yet to adopt them.

It should also be mentioned that the reason for low participation of some developing nations to do research in line with the current subject matter may be due to the fact that different countries give different priorities to the kind of research they do with respect to their peculiar societal, economic and environmental challenges facing them. Furthermore, another possible reason for low participation of developed/developing nations to do research in the discussed subject matter may be as a result of the fact that some countries may invest more in other sectors (e.g., ICT, finance, power, energy) other than in agriculture. In addition, the regulatory policies of some nations as it relates to agriculture may not encourage academic studies that will support the utilization of invertebrate species to promote agriculture.

The United States, China, Germany, the United Kingdom, Australia and Canada ranked in the top positions of countries involved in active research with the application of invertebrates for improving agriculture in terms of the amount/numbers of articles and citations (Tables 2 and 3). One main reason for any nation to be within the category of having high numbers of articles and citations in a given research area has been attributed to their financial support from various agencies including government agencies [40–43]. Again, increased participation in this type of research area could be ascribed to a potentially high rate of their involvement in both national and international networking with other research organizations, which are vital booster that improves research impact visibility and frequency of their study citations [33, 42, 44].

In line with our findings, the United States has shown immense contributions in numerous scientific research areas including in medicine, agriculture, microbiology, chemistry, computer science, engineering, environmental science and geography, among others [45–52]. A generally known factor that influences country's multinational collaborations is linked to authors' multiple research affiliations with several other institutions around the world [33]. Conversely, the relatively low involvements in research on invertebrates with regard to agriculture as portrayed from the current study by developing nations, especially African countries (with only South Africa being shortlisted in the top 25 nations), may be linked with the fact that most scientific studies done in these regions are customarily self-funded and most times they do not attract financial funding to support them [41, 51].

The 25 top listed nations with multiple collaboration (MCP) on invertebrates and agriculture studies showed that their networks were mostly among scientists from economically stable countries such as the United States, Germany, China, the United Kingdom and Spain (Table 2). This result is in line with other bibliometric studies who reported countries' collaboration and networking from among financially stable nations [43, 44]. It is generally noticed in bibliometric studies that collaborations between economically stable and developing nations are rare [33, 51, 53].

The current study also observed that among authors from Poland and the Czech Republic, collaboration pathways were solely from local research, with result of SCP (*n* = 11; *n* = 10) but with zero MCPs, respectively (Table 2). The lack of international collaboration conspicuously affected their (Poland and the Czech Republic) noninclusion among the 25 top listed highly cited nations in Table 3. On the other hand, collaboration by the authors in Denmark with just one incidence of MCP (*n* = 1) but with lesser article publications (*n* = 10) when compared to Poland (*n* = 11) and the Czech Republic (*n* = 10) was listed among the 25 most cited nations. This is indicative of the influence of international networking in attracting global citations to research articles. Global collaboration in scholarly research is also more significant because of the need for diffusion and exchange of ground-breaking ideas from the collaborating countries [33, 45]. Exchange programs and knowledge sharing in scientific studies from both intra- and international organizations among countries often afford more robust/fruitful opportunities and in pulling resources (human and financial) to carry out important research and breaching research gaps in any field of study [43].

There was a noticeable swing/switch in the rankings/ratings among the 25 top listed nations that are actively involved in the field of invertebrate and agriculture research when outputs were assessed based on total citation (TC) per nation (Tables 2 and 3). For example, China moved from the second position in Table 2 to the eighth position in Table 3. Similar ranking switch has also been reported in other bibliometric studies [33, 43, 44]. The closest interpretation with the swing/change in rankings when considering citation numbers to rate global author or nation's outputs simply shows its undependability as a correct benchmark for research productions. The amount of citations of articles does not truly mirror the article productivity of authors or nations. This is because according to Ref. [54] the lesser the amount of papers used for assessment, the more the influence of a few frequently cited articles. Several scholars are involved in self-citations, while several others make inaccurate citations in publications. This error of scholars promotes false quality and quantitative indexes of the aggregate citations (TC) of authors or countries. Invariably, due to how authors give false citations in the literature, this affects the true reflection of the productivity in terms of citations of individual authors [54].

Most scholarly articles that are online comprise a number of publication keywords to help other scholars with online searches and recognize some specific editors of a manuscript [55]. Author's keywords are used to cover areas of important subject matters of a scientific field, and they help interested readers of a particular manuscript to comprehend key concepts of the manuscript [56]. Keywords also help to project cutting hedge summary of any scholarly research paper [57]. Editors of the most journal sources usually demand list of author's keywords at the point of manuscript submission prior to the manuscript review process and prospective acceptance of such manuscript. This is indicative of the significance in the use of author's keywords to a manuscript being within the scope of a journal and its eventual acceptance or rejection by editors of journals [50].

In the present study, we used both the singular and the plural procedures of the author keywords to present the most occurring trail/trend on invertebrates and agriculture research. This approach is significant in helping readers to better comprehend the research emergence/evolution of the studied subject area [58]. The result from our search (WoS) portrays both the keywords and the keyword plus. This is essential because author keywords in any publication are a collection of terminologies given by writers/authors to showcase the content of the article, while keyword plus represents phrase/s appearing in the references of titles of a document but not in the titles of articles itself [59]. Between 1991 and 2022, an aggregate of 4145 author keywords and 4197 keyword plus terminologies were recovered from the archives on invertebrates and agriculture research (Table 1). Conversely, most of these authors' keywords and keyword plus including agriculture, biodiversity, arthropod/s, biological control, ecosystem services, sustainable agriculture and Carabidae, among others, are relevant to the research done on invertebrates as linked to agriculture (Table 4). Although it is alleged that invertebrates are seen as mere insects with insignificant contribution to the ecosystem [60], it is important to note that they have been used to achieve much (e.g., biocontrol, natural enemies, pest control and predators, among others) in agriculture and food production [5, 6, 39].

Figure 3 shows the foremost scientists (20 most relevant researchers) in the niche area of invertebrate and agriculture research. As observed from the result, Figure 3 describes the information of the 20 topmost relevant authors in the field of invertebrate and agriculture research, with the author named T. Tscharntke being ranked in the first position (*n* = 13). Judging from his scientific profile, he has a staggering h-index of 147 (with over 86,000 citations). Tscharntke is also a renowned and established researcher and a Professor of Agroecology (entomology, ecology) from the University of Göttingen, Germany. He has published extensively on research interest (insects and invertebrates) covering significant topics on biological control of weeds, biodiversity, insect pollination, pest management, ecosystem and insects as natural predators, among others. With the present *h*-index of Tscharntke as seen in his academic profile, it is a clear testament to his prolificacy in this research field. Basically, the h-index is used to assess the significance of articles in scientific research [61] and is also used to evaluate how productive and scientifically relevant researcher/s are within a research field via the amount of citations in their publications [62]. Other relevant scientists in this field of invertebrates as linked to agricultural research are S. Alles (*n* = 9), DW Crowder (*n* = 9), M.J. Samways (*n* = 8) and Y. Chen (*n* = 7). These researchers have equally published widely on the current studied topic with their academic profile having high citations and h-indexes. Scientists/researchers are rated via their scores in the h-index, which usually tallies with the amount (in volume) of articles produced or cited over a period of time. The h-index is estimated by using the *h*-algorithm (of research publications) on the minimum number of *h* times an article was cited [62]. Equally, the h-index assessment is a vital instrument in bibliometric evaluation, because it accurately replicates the impact of scholarly achievement a researcher/s has towards the pool of scientific knowledge [63]. The innovative research works of authors in this research domain are a testament to the significance of invertebrates in advancing agriculture. According to Refs. [4, 9], invertebrates are unique and naturally abundant organisms that can play vital roles in the entire agricultural systems, and they have the prospect to be utilized or manipulated for the purpose of improving agriculture.

With reference to journal sources, they are regarded as essential aspects in bibliometric studies to be considered in order to describe the prospect of research spread of a particular scientific research domain [64]. The 25 most impactful journal sources in the present discussed subject matter in Table 5 show a reasonable correlation that they are credible outlets responsible for publishing scholarly information/findings that are in line with invertebrates and agricultural research studies. From Table 6, the information for the most relevant research institutions with the uppermost amount of research outputs in invertebrate and agriculture niche showed that the United States of America led the chart with institutions doing research in this field. Other previous bibliometric studies have also observed similar findings that institutions from the United States make significant contributions to the body of scientific knowledge in several other research areas [41, 45, 46, 50].

With respect to the global citation of an article and its relevance in the world stage, the general world indices of citing a particular paper indicate the number of citation that article receives over a time span and how many times the said article was downloaded by other people. In addition, the global citation relies on the academic impact of the citing article rather than just its popularity. An academic publication cited by a high impact factor journal attracts the attention of several other scientists, while the number of citations that a publication draws to itself indicates its global impact. The importance of an article in the global space is mostly assessed by the number of times it was cited [65]. However, the article impact grows significantly relevant to more citations [66].

The top 20 globally cited articles that were assessed based on TCs per year (TC/Year) and TCs from invertebrate and agriculture studies from 1991 to 2022 are presented in Table 7. These scholarly papers were published in the journal Ecological Application (TC = 3900), Journal of Pest Biochemistry and Physiology (TC = 696), Nature (TC = 527), Journal of Applied Ecology (TC = 466) and Biocontrol Journal (TC = 425), among others. The findings from these aforementioned top valued articles spread across different topics on the use of invertebrates to improve agriculture, including ‘The efficacy of invertebrates (insect, mite and tick) control products via effective resistance management for sustainable agriculture and improved public health—Sparks and Nauen [67]'. Likewise, another top rated article described the effects of using ‘invertebrates as biological control to improve agricultural production-van Lenteren et al.' [9]. Again, another top ranked study reported the findings of ‘The role of earthworms for assessment of sustainability and as bio-indicators—Paoletti [68]'. Although several articles may fall within the ranks of being one of the most cited about a particular subject matter, it should be stated that an increase in paper citations sometimes may come with negative criticism of the article's research findings and content of the study [69].

With respect to global networking in research, this is a crucial criterion employed in bibliometric studies. It is used to know how to move scientific investigations about a particular research niche forward, as it improves partnership among researchers of similar research interest globally. Networking also permits both inter- and intradisciplinary exchange of intellectual ideas from different cadres among researchers involved in similar research work [70]. Scientific networking and collaboration also improve the quality of outputs of the research study [71]. Other important advantages of research networking include the publication of novel articles, discovering of ground-breaking findings, exchange of intellectual human capacity, funds/resources availability and utilization of proficient facilities, among others [72]. The result of nation's networking and collaborations is shown in Figure 4 using different colours to demonstrate their groupings in line with their co-operation. In all, five groups were showcased in the diagram with Iran found in an isolated group (with an orange colour) from all other countries. The reason for this isolation may not be clearly known but may not be unconnected to the fact that they are not among the topmost relevant nations as seen in Tables 2 and 3.

Consequently, the node displaying each country and the strokes/lines linking the countries together have different thickness levels and magnitudes. This is indicative of how significant and strong the partnership among these nations is. The names of the countries presented in Figure 4 are in small letters by default from the RStudio software package. RStudio software often presents countries networking in small lettering [73]. The United States showed that it had the most influence and networks with other nations (with the largest node). This finding is true with other authors reporting similar findings [51, 53, 74]. The function evaluations and good-of-fit indicated that the findings on invertebrates and agriculture research support earlier studies, which agreed with the fact that invertebrate activities including, but not limited to, pollination, dung decomposition, pest control and nutrient cycling, among others, enhance agriculture [5, 6, 12, 75]. However, many different management interventions and practices on farmed landscapes have been reported to have profound implications on the abundance, diversity, species richness and composition of invertebrate populations that may negatively affect agricultural productivity and sustainability [4, 76]. In addition, the growing global agricultural intensification and environmental pollution are known to have detrimental effects on biodiversity of invertebrates [77, 78]. It leads to a rapid loss of invertebrate diversity, both above- and belowground. There are many reasons for this loss (economically important invertebrates), including increasing homogenization of agricultural systems, excessive soil disturbance caused by continuous tillage, monocultures and use of agrochemicals [4, 76].

The United States had the highest number of collaborations/networks (*n* = 67), followed by China (*n* = 32), Germany (*n* = 31) and the United Kingdom (*n* = 31). Several literature studies show that the United States as a nation among other developed nations conducts several research studies in line with the discussed subject matter [76, 79]. Nation collaboration networks are assessed using RStudio (R programming language) where the magnitude of the circle signifies the number of articles published by the country. Meanwhile, the link/association between two circles shows the collaboration among them and link strength is the total sum of articles they have published together. The total link strength of a circle represents the total number of collaborations that a nation has made with other countries worldwide.

Furthermore, the result in Figure 6 investigates the authors' keywords by adopting the thematic evaluation map to explain how highly relevant author's keywords faired over the years. This kind of bibliometric assessment was earlier explained by Ref. [80] to report relevant growing issues on a scientific subject matter. In the present study, the thematic evaluation map presents four key themes founded on the authors' keywords collaboration/network breakdown and clustering (Figure 6). Firstly, the top-right quadrant is known to be the motor theme, and it signifies the high centrality and concentration keywords of the studied subject matter. ‘Molluscicide', ‘pesticide', ‘coral', ‘insecticides', ‘agriculture', ‘management' and ‘insects' are the keywords that appear to be the most developed in the research area of invertebrates and agriculture. Secondly, the top-left quadrant known as the niche theme contains some trending the themes such as ‘growth', ‘mollusc', ‘coral reefs', ‘predator', ‘chitin' and ‘chitosan'. This second theme represents the ‘high' centrality and relevancy to the studied research of invertebrates and agriculture, although they have not yet been properly developed.

Conversely, in the bottom-right theme (Figure 6), terminologies such as ‘nitrogen', ‘eutropication', ‘resistance', ‘pesticides', ‘toxicity', ‘earthworm', carabidae', ‘soil', ‘araneae', ‘ecosystem services', ‘pest control', ‘biodiversity', ‘arthropods' and ‘biological controls' tend to have a high focus among scientists, but they are still not developed and centralized. Lastly, the bottom-left quadrant known as the emerging or declining theme comprises challenges-associated author keywords including ‘biocontrol', ‘diet', ‘climate change', ‘mollusca' and ‘development'. This theme shows that the challenge of invertebrate and agriculture research is still emerging and not properly developed, but it has little significant external links with the other keywords. Recently, member nations of the Food and Agriculture Organization (FAO) of the United Nations have identified the excessive use of chemical control mechanisms (such as pesticides and antibiotics) as the most impactful practice that has been driving the loss of soil biodiversity in the last 10 years [81]. This revelation has increased the need for more research in the studied subject matter. Conversely, due to research investigations, several soil invertebrates are known to be involved in controlling agricultural pests. For instance, nematodes and mites are utilized for targeting disease-related bacteria in crops [82, 83]. In addition, predators and parasitoids (e.g., parasitic wasps and beetles) prey/feeds on arthropods that interfere with crop production [84], while herbivorous soil insects feed on the seeds of undesirable plants selectively over crop seeds, thereby reducing the spread of aggressive weeds that can negatively affect agricultural produce [85]. Altarturi et al. [52] also used the thematic evolution map to report the emergence of advance technology in advancing agricultural e-commerce, another emerging and interesting field in agriculture.

Over the years, there has been an increase in research numbers in the advancement of studies on the use of invertebrates to promote agriculture (Figure 2). One motivating force behind this may be attributed to the fact that there are several diverse invertebrates being naturally abundant in our ecosystem and they are significant agricultural biodiversity involved in regulating the strata and primary functions of the natural ecosystem [3, 8]. They are also very essential in the natural food webs as they form part of the ecosystem engineers linked with agriculture and food production [6].

To date, our paper seems to be the first bibliometric investigation that evaluated the research publications of scientific peer-reviewed findings on invertebrates and agriculture at a global stage. Subsequently, we are cognizant of the fact that there might be some constraints/limitations to the current work including but not restricted to the following:

a. The possibility of omitting some publications that we might not have added in the evaluation of invertebrates and agriculture or its related words during the data retrieval since we used only WoS.
b. Our findings may also be limited since we did not add articles on invertebrates and agriculture that were in nonindexed journals, including those published online in other languages and non-English journals.
c. The current research result might also be constrained due to the removal of other article types including technical notes, conference proceedings, review articles, meeting abstracts and note papers.

### 4.1. Study Limitations

Regardless of the several benefits of this type of analysis (bibliometric), it is essential to acknowledge some limitations that are related to the current study. Published articles related to invertebrates in the context of agriculture were evaluated using only WoS archive to accommodate an enormous coverage of the required articles. However, it is not unlikely that some other articles published in journals that are not indexed in the chosen database (i.e., WoS) were excluded. Thus, the results of the present study may not have represented the whole publications available on the studied topic. Additionally, the journals assessed in this study were constrained to the ones written solely in English language, without considering others written in other internationally recognized languages. Hence, it is suggested that future studies in this niche area (invertebrates in the context of agriculture) should consider all the enumerated limitations to allow for a more accurate inclusiveness.

## 5. Conclusion

The current bibliometric study revealed a worldwide spread in the use of invertebrates for improving agriculture, with majority of the research studies carried out in high-income nations. The study also revealed limited collaboration/networking among high-income and developing countries. The low research publications in developing nations on the current studied topic also echoed similar results in other studied scholarly research fields in bibliometric.

From our findings using the thematic evolution and literature results, invertebrate research in the context of agriculture is tending towards biogeography, farmland biodiversity, insecticides and organic agriculture, which are of immense importance to scientists and researchers in this research domain, thus signifying the direction/path of future research.

Again, we propose that a more robust and holistic studies steered by meta-analysis narrative (in the future) should be done to focus on more emerging themes and trending research directions/pathways on the use of invertebrates to promote agriculture. More especially these studies (e.g., the use of genetic engineering in economically valuable insects) should be carried out in developing nations where funds and modern facilities are limited to do research. This is because most developing countries face food insecurity on a large scale; hence, adopting new innovative strategies (that is workable within the environment and geographical area) in the use of invertebrates for agricultural improvements will increase food sustainability and security.

## Figures and Tables

**Table 1:** Information summary on articles retrieved on the study trend on invertebrates and agriculture from the WoS database.

Description	Results
Main information about data	
Timespan	1991:2022
Sources (journals, books, etc.)	523
Documents	1201
Annual growth rate %	11.03
Document average age	8.63
Average citations per doc	31.22
References	56,346
Document contents	
Keywords plus (ID)	4145
Author's keywords (DE)	4197
Authors	
Authors	4985
Authors of single-authored docs	69
Authors collaboration	
Single-authored docs	71
Coauthors per doc	4.79
International coauthorships %	32.22
Document types	
Article	1201

**Table 2:** Twenty-five most productive countries in invertebrates and agriculture research based on the number of articles based on corresponding authors.

S/N	Country	Articles	% of article	SCA	MCA	Frequency	MCP_ratio
1	United States	312	25.97	245	67	0.26	0.215
2	China	88	7.32	56	32	0.073	0.364
3	Germany	75	6.24	43	32	0.062	0.427
4	United Kingdom	70	5.82	39	31	0.058	0.443
5	Australia	60	4.99	35	25	0.05	0.417
6	Canada	46	3.83	36	10	0.038	0.217
7	Italy	46	3.83	34	12	0.038	0.261
8	Brazil	45	3.74	38	7	0.037	0.156
9	Spain	37	3.08	27	10	0.031	0.27
10	France	33	2.74	19	14	0.027	0.424
11	South Africa	26	2.16	19	7	0.022	0.269
12	India	22	1.83	15	7	0.018	0.318
13	New Zealand	18	1.49	15	3	0.015	0.167
14	Sweden	18	1.49	9	9	0.015	0.5
15	Japan	16	1.33	10	6	0.013	0.375
16	Switzerland	16	1.33	6	10	0.013	0.625
17	Argentina	12	0.99	9	3	0.01	0.25
18	Israel	12	0.99	9	3	0.01	0.25
19	Egypt	11	0.91	10	1	0.009	0.091
20	Poland	11	0.91	11	0	0.009	0
21	Chile	10	0.83	7	3	0.008	0.3
22	Czech Republic	10	0.83	10	0	0.008	0
23	Denmark	10	0.83	9	1	0.008	0.1
24	Korea	10	0.83	6	4	0.008	0.4
25	Netherlands	10	0.83	5	5	0.008	0.5

Abbreviations: MCA = multiple-country articles, SCA = single-country articles.

**Table 3:** Most relevant countries in invertebrates and agriculture research based on the number of total article citations.

S/N	Country	TC	Position	Average article citations
1	United States	14,113	1^st^	45.20
2	Germany	3686	2^nd^	49.10
3	United Kingdom	3117	3^rd^	44.50
4	Australia	1607	4^th^	26.80
5	Canada	1419	5^th^	30.80
6	Switzerland	1208	6^th^	75.50
7	France	1152	7^th^	34.90
8	China	1057	8^th^	12.00
9	Italy	995	9^th^	21.60
10	Spain	682	10^th^	18.40
11	Brazil	676	11^th^	15.00
12	Belgium	654	12^th^	72.70
13	New Zealand	643	13^th^	35.70
14	Netherlands	545	14^th^	54.50
15	Sweden	503	15^th^	27.90
16	South Africa	342	16^th^	13.20
17	India	285	17^th^	13.00
18	Japan	274	18^th^	17.10
19	Denmark	254	19^th^	25.40
20	Greece	223	20^th^	27.90
21	Israel	205	21^st^	17.10
22	Malaysia	202	22^nd^	28.90
23	Norway	201	23^rd^	100.50
24	Turkey	198	24^th^	19.80
25	Portugal	196	25^th^	21.80

Abbreviation: TCs = total citations.

**Table 4:** Twenty most relevant words used by the authors in invertebrates and agriculture research.

S/N	Author's keywords (DE)	Proportion	Keywords plus (ID)	Proportion
1	Agriculture	141	Biodiversity	169
2	Biodiversity	66	Diversity	152
3	Arthropod/s	66	Management	145
4	Biological control	46	Agriculture	125
5	Ecosystem services	46	Abundance	94
6	Sustainable agriculture	33	Arthropods	78
7	Carabidae	31	Conservation	77
8	Conservation	31	Biological control	73
9	Organic agriculture	26	Communities	65
10	Organic farming	26	Natural enemies	61
11	Management	25	Ecosystem services	50
12	Pesticides	22	Habitat	48
13	Soil	22	Land-use	48
14	Pest control	21	Populations	47
15	Diversity	20	Growth	46
16	Insects	18	Coleoptera	45
17	Integrated pest management	17	Systems	42
18	Pest	17	Landscape	39
19	Predators	17	Impacts	37
20	Agroecology	16	Predators	37

**Table 5:** The 25 most relevant journal sources in invertebrates and agriculture research based on the number of articles produced.

S/N	Sources	Articles	% of articles	Position
1	Agriculture ecosystems and environment	69	5.74	1^st^
2	Journal of AOAC international	27	2.24	2^nd^
3	PLoS One	25	2.08	3^rd^
4	Journal of Applied Ecology	23	1.91	4^th^
5	Insects	21	1.74	5^th^
6	Environmental Entomology	19	1.58	6^th^
7	Biological Control	16	1.33	7^th^
8	Ecological Applications	16	1.33	7^th^
9	Marine Pollution Bulletin	15	1.24	8^th^
10	Science of the Total Environment	15	1.24	8^th^
11	Pest Management Science	13	1.08	9^th^
12	Agronomy-Basel	11	0.91	10^th^
13	Proceedings of the National Academy of Sciences of the United States of America	11	0.91	10^th^
14	Biodiversity and Conservation	10	0.83	11^th^
15	PeerJ	10	0.83	11^th^
16	Renewable Agriculture and Food Systems	10	0.83	11^th^
17	Applied Soil Ecology	9	0.74	12^th^
18	Basic and Applied Ecology	9	0.74	12^th^
19	Biological Conservation	9	0.74	12^th^
20	Crop Protection	9	0.74	12^th^
21	Environmental Science and Pollution Research	9	0.74	12^th^
22	Journal of Food Protection	9	0.74	12^th^
23	Journal of Insect Conservation	9	0.74	12^th^
24	Journal of Economic Entomology	8	0.66	13^th^
25	Scientific Reports	8	0.66	13^th^

**Table 6:** The 24 topmost global relevant institutes on invertebrates and agriculture studies with over 15 research publications.

S/N	Affiliation	Nations	Articles	Rankings
1	Michigan State Univ	United States	56	1^st^
2	Univ Calif Davis	United States	39	2^nd^
3	Univ Florida	United States	38	3^rd^
4	Univ Queensland	Australia	35	4^th^
5	Washington State Univ	United States	35	4^th^
6	Univ Hawaii Manoa	United States	33	5^th^
7	Univ Georgia	United States	32	6^th^
8	Univ Wisconsin	United States	32	6^th^
9	Penn State Univ	United States	30	7^th^
10	Univ Hawaii	United States	30	7^th^
11	Univ Gottingen	Germany	29	8^th^
12	Swedish Univ Agr Sci	Sweden	26	9^th^
13	Aarhus Univ	Denmark	24	10^th^
14	Stanford Univ	United States	24	10^th^
15	Univ Calif Berkeley	United States	24	10^th^
16	Univ Fed Vicosa	Brazil	22	11^th^
17	Univ Ghent	Belgium	22	11^th^
18	Lund Univ	Sweden	20	12^th^
19	Ohio State Univ	United States	20	12^th^
20	Univ Guelph	Canada	18	13^th^
21	James Cook Univ	Australia	17	14^th^
22	Stellenbosch Univ	South Africa	17	14^th^
23	Oregon State Univ	United States	16	15^th^
24	Univ Kassel	Germany	16	15^th^

**Table 7:** Top 20 most globally cited documents on invertebrates and agriculture research.

S/N	Author first name and year of publication	Journal name	DOI	Total citations	TC per year	Normalized TC
1	Carpenter S.R., 1998	Ecol appl	10.2307/2641247	3900	150.00	11.63
2	Sparks T.C., 2015	Pest biochem physiol	10.1016/j.pestbp.2014.11.014	696	77.33	18.85
3	Seibold S., 2019	Nature	10.1038/s41586-019-1684-3	527	105.40	22.19
4	Benton T.G., 2002	J appl ecol	10.1046/j.1365-2664.2002.00745.x	466	21.18	8.19
5	van Lenteren J.C., 2018	Biocontrol	10.1007/s10526-017-9801-4	425	70.83	16.50
6	Kremen C., 2012	Ecol soc	10.5751/ES-05035-170440	380	31.67	11.92
7	Rusch A., 2016	Agric ecosyst environ	10.1016/j.agee.2016.01.039	317	39.63	13.38
8	Drinkwater L.E., 1995	Ecol appl	10.2307/2269357	316	10.90	3.82
9	Werling B.P., 2014	Proc natl acad sci USA	10.1073/pnas.1309492111	314	31.40	7.24
10	Dermauw W., 2013	Proc natl acad sci USA	10.1073/pnas.1213214110	289	26.27	6.52
11	Tscharntke T., 2008	Ecology	10.1890/07-0455.1	285	17.81	6.69
12	Paoletti M.G., 1999	Agric ecosyst environ	10.1016/S0167-8809(99)00034-1	283	11.32	3.31
13	Martin E.A., 2019	Ecol lett	10.1111/ele.13265	261	52.20	10.99
14	Mooney H., 2009	Curr opin environ sustain	10.1016/j.cosust.2009.07.006	260	17.33	6.20
15	Evans J.D., 2013	J hered	10.1093/jhered/est050	245	22.27	5.53
16	Meehan T.D., 2011	Proc natl acad sci USA	10.1073/pnas.1100751108	240	18.46	5.86
17	Schweiger O., 2005	J appl ecol	10.1111/j.1365-2664.2005.01085.x	231	12.16	4.52
18	Davidson E.A., 2004	Ecol appl	NA	225	11.25	3.52
19	Duelli P., 1999	Agric ecosyst environ	10.1016/S0167-8809(99)00029-8	224	8.96	2.62
20	Thaler J.S., 1999	J chem ecol	10.1023/A:1020840900595	218	8.72	2.55

## Data Availability

The data will be made available on request.
